# Exome Sequencing Identified Molecular Determinants of Retinal Dystrophies in Nine Consanguineous Pakistani Families

**DOI:** 10.3390/genes13091630

**Published:** 2022-09-10

**Authors:** Raeesa Tehreem, Iris Chen, Mudassar Raza Shah, Yumei Li, Muzammil Ahmad Khan, Kiran Afshan, Rui Chen, Sabika Firasat

**Affiliations:** 1 Department of Zoology, Faculty of Biological Sciences, Quaid-i-Azam University, University Road, Islamabad 45320, Pakistan; 2 Department of Molecular and Human Genetics, Baylor College of Medicine, Houston, TX 77030, USA; 3 Gomal Center of Biochemistry and Biotechnology, Gomal University, Dera Ismail Khan 29111, Pakistan; 4 Department of Human Genetics, Sidra Medicine, Doha P.O. Box 26999, Qatar

**Keywords:** retinaldystrophies, night blindness, homozygous sequence variants, autosomal recessive

## Abstract

Inherited retinal dystrophies (IRDs) are a heterogeneous group of degenerative disorders of the retina. Retinitis Pigmentosa (RP) is a common type of IRD that causes night blindness and loss of peripheral vision and may progress to blindness. Mutations in more than 300 genes have been associated with syndromic and non-syndromic IRDs. Recessive forms are more frequent in populations where endogamy is a social preference, such as Pakistan. The aim of this study was to identify molecular determinants of IRDs with the common presentation of night blindness in consanguineous Pakistani families. This study included nine consanguineous IRD-affected families that presented autosomal recessive inheritance of the night blindness phenotype. DNA was extracted from blood samples. Targeted exome sequencing of 344 known genes for retinal dystrophies was performed. Screening of nine affected families revealed two novel (c.5571_5576delinsCTAGATand c.471dup in *EYS* and *SPATA7* genes, respectively) and six reported pathogenic mutations (c.304C>A, c.187C>T, c.1560C>A, c.547C>T, c.109del and c.9911_11550del in *PDE6A*, *USH2A*, USH2A, *NMNAT1*, *PAX6* and *ALMS1* genes, respectively) segregating with disease phenotype in each respective family. Molecular determinants of hereditary retinal dystrophies were identified in all screened families. Identification of novel variants aid future diagnosis of retinal dystrophies and help to provide genetic counseling to affected families.

## 1. Introduction

Inherited retinal dystrophies (IRDs) are degenerative eye disorders causing substantial loss of vision and even blindness [1]. The most common inheritable retinal dystrophy is retinitis pigmentosa (RP), which is characterized by the degradation of photoreceptors, predominantly rods and secondarily tightly packed cones [2]. In the initial stage of disease, night vision is reduced, followed by loss of peripheral vision in the diseased individuals [3]. Different values of prevalence are reported from various regions worldwide; from 1:372 in rural regions of South India [4] to 1:9000 in Korea [5]. RP can be present in both non-syndromic and syndromic forms. In non-syndromic forms, it can be inherited in autosomal pattern (dominant/recessive), X-linked pattern (dominant/recessive), mitochondrial inheritance or sporadic cases [6]. Syndromic forms of RP such as Bardet–Biedl syndrome (MIM no. 209900) and Usher syndrome (MIM no. 276901) are associated with extra ocular abnormalities and are reported in almost 20–30% cases [3].

Significant overlap at both the clinical and molecular level is observed between different forms of IRDs. For example, early onset RP can be detected at the age of 2 years and could overlap with Leber’s congenital amaurosis (LCA) (MIM # 204000) [2], but in most cases symptoms manifest in adolescence [1].LCA is a severe autosomal recessive retinopathy [7] that presents in children by birth or in early years of life [8]. Nyctalopia is the earliest clinical symptom experienced by a patient, followed by photophobia, loss of visual acuity, abnormal fundus, bone spicules deposition, tunnel vision, waxy pallor of optic nerve, attenuated retinal vessels and abnormal or absent a- and b- wave amplitudes in electroretinogram (ERG) [9]. Heterogeneity of clinical findings in IRDs is partially due to the involvement of different genes and alleles. Until now, more than 80 genes have been associated with non-syndromic forms of RP, which function in diverse pathways [1]. Similarly, fourteen genes are reported to cause LCA, encoded proteins which are involved in the development and physiology of the visual pathway such as CRB1 (photoreceptor morphogenesis), CRX (retinal development), MERTK (RPE phagocytosis), RDH12 (retinal reductase), LRAT (Lecithin Retinol Acyltransferase), RPE65 (vitamin A metabolism) RPGRIP1L (cilium formation) [10,11] NMNAT1 (protein multimerization) [12], GUCY2D (Guanylate Cyclase 2D) and AIPLI (phototransduction) [13]. In families of Pakistani origin, most of the mutations in IRDs patients are reported in genes encoding the PDE6A, PDE6B, ABCA4, RHO, SPATA7, TULP1 and RP1 protein [14].

Syndromic forms of IRDs also show heterogeneity, as 15 genes are associated with Usher syndrome [15] and 20 genes (BBS1-BBS20) are reported for Bardet–Biedl syndrome [16]. Usher syndrome (USH) is an autosomal recessive form of RP that is accompanied by hearing impairment and functional abnormality of the vestibular system [17]. Characterization into subtypes USH type I (MIM no. 276900), USH type II (MIM no. 276901) and USH type III (MIM no. 276902) is based on intensity of hearing loss, onset of RP and presence or absence of vestibular dysfunction. Although USH is heterogeneous [18], the *USH2A* gene is the most frequently mutated and accounts for 74% to 90% cases of UHS type II [19]. Proteins coded by Usher genes are usually expressed in transmembrane regions, scaffolding proteins and in motor transport. Predominantly, nonsense and splice site mutations result in syndromic conditions in affected cases [20,21].

Prevalence of consanguinity is very high in Pakistan [4], resulting in the high prevalence of recessive forms of IRD. Due to the high genetic heterogeneity, the conventional molecular diagnostic method, e.g., Sanger’s sequencing, is not cost effective. Therefore, the current study was designed to investigate the molecular determinants of IRD phenotypes in the Pakistani population using Next Generation Sequencing (NGS) technology to perform panel sequencing targeting exons of 344 known inherited retinal disease genes. In this study, nine consanguineous families suffering from IRDs, each having at least three or more affected individuals, were analyzed.

## 2. Materials and Methods

### 2.1. Enrollment of Patients for Study

**Figure 1:**
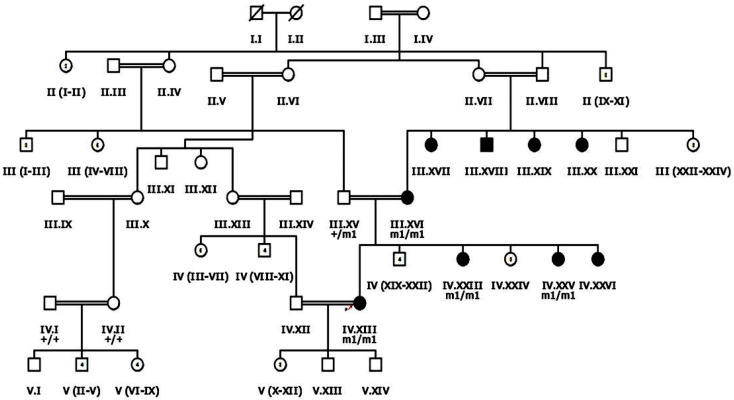
Pedigree drawing of inherited retinal dystrophy family, i.e., RP107 showing autosomal recessive pattern of phenotype in which the novel disease-causing variant in the *EYS* gene was detected. Squares and circles denote males and females, respectively. Filled symbols show affected, while unfilled symbols show unaffected individuals. Double lines indicate consanguineous union. M1/m1 refers to the homozygous disease-causing variant, whereas m1/+ and +/+ refers to heterozygous carrier and homozygous normal status, respectively.

**Figure 2:**
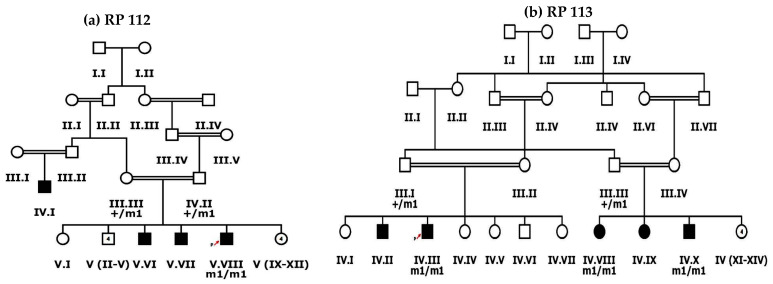
Pedigree drawings of inherited retinal dystrophies families, i.e., (**a**) RP112 and (**b**) RP113 showing autosomal recessive pattern of phenotype in which the novel disease-causing variant in the *SPATA7* gene was detected. Squares and circles denote males and females, respectively. Filled symbols show affected, while unfilled symbols show unaffected individuals. Double lines indicate consanguineous union. m1/m1 refers to the homozygous disease-causing variant, whereas m1/+ refers to heterozygous carrier status.

Nine familial cases of inherited retinal dystrophies, clinically assessed by ophthalmologists, were recruited from Dera Ismail Khan, Khyber Pakhtunkhwa (KPK) Pakistan following the Declaration of Helsinki [22,23]. Approval was received from the Bioethical review Committee (BEC) of Quaid-i-Azam University (QAU) Islamabad before beginning the study. Blood samples were collected from patients and phenotypically normal family members. Clinical data of each proband, family history of disease and data regarding any other disorder in family member/s were also recorded after informed consent (Table 1). Inclusion criteria for patients recruited after ophthalmological assessment was based on the symptom of night blindness and recessive mode of inheritance. Haplopainter (http://haplopainter.sourceforge.net/index.html (accessed on 5 April 2021)) [24] was used to draw pedigrees of all families, with a unique identification number given to each family (Figure 1 and Figure 2 and Supplementary Figures S1 and S2).

### 2.2. Sample Collection, DNA Extraction and Targeted Exome Sequencing

Peripheral blood samples were collected in 5ml ethylene diamine tetra acetic acid (EDTA) vacutainers and stored at −20 °C. To extract genomic DNA from blood samples, a non-organic method of DNA extraction described by Kaul et al., 2010 [25] was used. Nano Drop (Thermo Scientific NanoDrop spectrophotometers, Waltham, MA, USA) was performed to quantify DNA and assess its purity at the Department of Zoology, Quaid-i-Azam University, Islamabad, Pakistan. For NGS panel capture sequencing, DNA samples were shipped to Baylor College of Medicine, USA. The sequencing library was generated using KAPA HyperPrep Kit (Roche, Basel, Switzerland) following the manufacturer’s protocol, then pooled together for targeted enrichment of a panel of 344 known and candidate inherited retinal diseases related genes (Supplementary Table S1) with the SureSelect Target Enrichment System for the Illumina Platform (Agilent, Santa Clara, CA, USA) [23]. Captured DNA was quantified and sequenced using a Novaseq 6000 (Illumina, San Diego, CA, USA). All these procedures were conducted at the Functional Genomics Core at Baylor College of Medicine, USA.

### 2.3. Bioinformatics Analysis

Sequencing data was processed following the previous methods [23]. Briefly, the fastq read files were aligned to the hg19 human reference genome using bwa. Aligned reads were recalibrated and realigned using GATK. Variants were called using GATK. All variants were filtered against dbSNP, 1000 Genome Projects, gnomAD V2.1.1 and BCM_HGSC internal database. Variants passing the filtering steps were evaluated according to the ACMG standards and guidelines for variant interpretation. Previously reported pathogenic mutations were identified through searching HGMD, ClinVar and LOVD databases. Novel variants were evaluated for their potential impact on protein function using various in silico tools. Nonsense, frame-shift and splicing mutations were classified as likely loss-of-function alleles. Missense variants were evaluated based on features such as sequence conservation and in silico predictions.

### 2.4. Sanger’s Validation and Segregation Test

Each candidate pathogenic variant was validated via direct Sanger’s sequencing. Primers were designed using the Primer3 software (https:P//bioinfo.ut.ee/primer3-0.4.0/ (accessed on 15 September 2021)) to amplify the region containing the variant with at least 50 flanking base pairs. Sanger’s sequencing and segregation test was performed on the amplified fragments for the affected proband, other affected members and non-affected members of each family based on availability of DNA samples. For the confirmation of previously reported large deletion, primers were adopted from Nikopoulos et al., 2015 [26] to exactly define the break points for the deleted region in a control and an affected individual of family RP109. Furthermore, for segregation testing of a large deletion identified in RP109, primers were designed using the Primer3 software to amplify the deleted region in a control and affected samples (Supplementary Table S2).

## 3. Results

In the current study, nine large multigenerational consanguineous families with inherited retinal dystrophies were enrolled from different regions of Dera Ismail Khan, KPK, Pakistan. Each enrolled family has multiple affected individuals (Figure 1 and Figure 2 and Supplementary Figures S1 and S2).

Families RP105 and RP109 had three affected individuals, while RP112 had four, RP101, RP110 and RP113 had five, RP102 and RP106 had six and RP107 had nine affected members. The phenotypic onset of disease in proband of enrolled families ranged from birth to the second decade of life (Table 1). In families RP101, RP106, RP110, RP112 and RP113, probands (III.IV, V.II, V.IV, V.VIII and IV.III, respectively) were affected by birth (Figure 2 and Supplementary Figure S1). Individual IV.I in family RP102 and IV.I in family RP109 were affected in the first decade of life, while V.IV in family RP105 and IV.XIII in family RP107 were affected in the second decade of life (Figure 1 and Supplementary Figures S1 and S2). Average age of proband at the time of enrollment was 28 years (Table 1).

**Figure 3:**
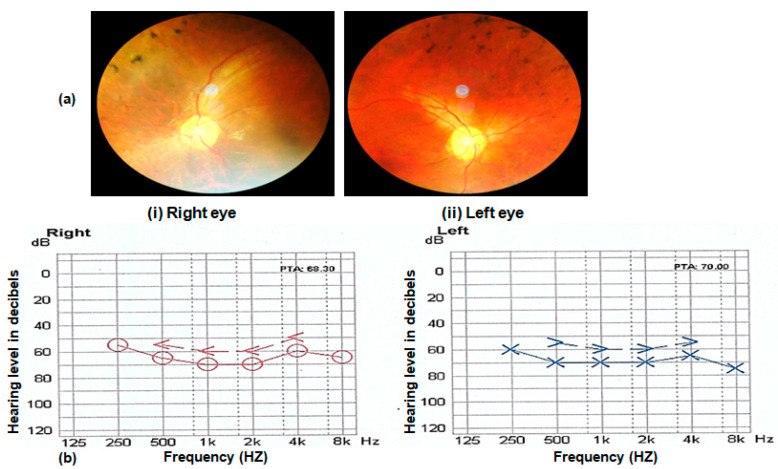
(**a**) Fundus photograph of left and right eye of an affected individual (V.II of RP105) showing pale optic disc, bony spicules and thin blood vessels characteristic of RP phenotype. (**b**) Audiometry report of an affected individual (V.II of RP105) performed at 14 years. The x-axis on graphs shows the frequency in hertz, and the y-axis shows hearing level in decibels (dB). Note the hearing loss in the range of 50–80 dB in both ears of the Usher patient.

In addition to the retinal phenotypes shared by affected individuals of all families, such as night blindness, poor day vision and photosensitivity, three (RP102, RP105 and RP109) out of nine families were suffering from syndromic disease. The affected individuals of families RP102, RP105 and RP109 were also suffering from progressive hearing loss. Nystagmus is observed in the affected individuals of family RP109. Fundus examination of probands of all affected families showed typical symptoms, including bone spicule formation, waxy pale discs and attenuation of vessels. Fundus photograph and audiometry findings of one affected individual (V.II) of family RP105 are shown in Figure 3 (Figure 3a,b).

**Figure 4:**
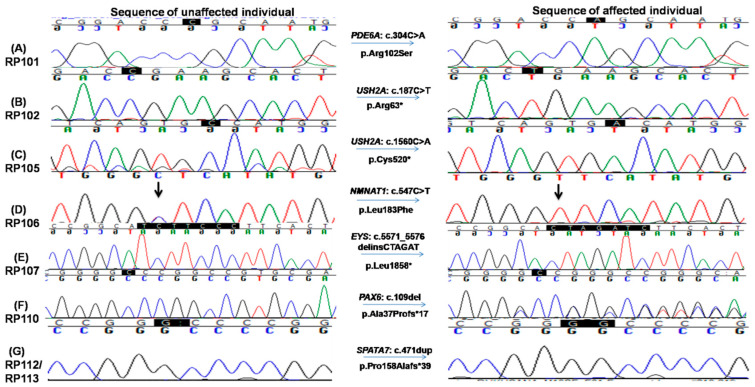
Chromatograms of known and novel disease causing variants (single nucleotide substitution/s, deletion, duplication and small INDELs) identified in this study, i.e., (**A**) c.304C>A in RP101, the homozygous sequence is shown on the left and homozygous mutated on the right (proband III.IV), (**B**) c.187C>T in RP102 showing homozygous normal on the left side and homozygous mutated on the right (proband IV.I) and (**C**) c.1560C>A in RP105 the homozygous wild-type sequence is shown on the left, whereas homozygous mutated on the right side (proband V.IV). (**D**) c.547C>T in RP106, homozygous mutated sequence is shown on the right, while homozygous normal sequence is present on the left (proband V.II); (**E**) c.5571_5576delinsCTAGAT in RP107 is shown on the right, whereas the sequence of normal individuals is shown on the left side; (**F**) c.109del in RP110 showing heterozygous mutated sequence on the right while wild-type sequence is shown on the left; and (**G**) c.471dup in RP112 and RP113, heterozygous mutated sequence is shown on the right, while normal sequence is on the left side.

To identify the underlying mutations of these families, NGS capture panel sequencing was performed on the proband of each family to screen for mutations in known inherited retinal diseases related genes. A total of 8 diverse types of pathogenic mutations from 7 different genes were identified, including 2 missense, 3 nonsense, 2 frameshift indel and 1 large deletion (Table 2, Figure 4 and Supplementary Figure S2).

Among them, six pathogenic variants were previously reported, while the remaining two were novel. Consistent with the nature of consanguineous families, 7 of 8 variants are homozygous mutations in recessive genes. Surprisingly, a heterozygous pathogenic mutation in *PAX6* is identified in family RP110 (Figure 4F).

The proband III.IV from the RP101 family was 41 years old at the time of enrollment for this study. Detailed interview of the patient revealed onset of RP phenotype in early childhood with initial complaints of poor night vision and then progressive loss of day vision. Mutation screening identified a homozygous missense variant c.304C>A (p.Arg102Ser) in the *PDE6A* gene which affects a highly conserved residue (Figure 4A). This variant is rare, with a population frequency of 0.016% according to the gnomAD database. Consistently, segregation testing in five affected family members indicated that mutation co-segregates with the retinal disease phenotype.

Two homozygous nonsense mutations in the *USH2A* gene, i.e., c.187C>T: p.Arg63* and c.1560C>A: p.Cys520*, were found in RP102 and RP105, respectively (Figure 4B,C). Consistent with the molecular diagnosis, patients from both families exhibit RP and hearing loss. The p.Arg63* variant in RP102 is a reported pathogenic mutation with a low population frequency of 0.001% in gnomAD. Segregation testing was carried out for the three affected and five unaffected family members, and perfect segregation between the mutations with the disease phenotype was observed (Figure 4B). One RP-affected (V.I) and two unaffected (V.III and a paternal aunt of IV.I) family members had mental retardation phenotype, which needs further investigation to find its causes. The stop-gain mutation p.Cys520* identified in family RP105 suffering from Usher syndrome is also previously reported [30,32] and likely to be pathogenic as it is predicted to result in a truncated protein that is approximately 10% of the normal size. As a premature truncation, the mRNA is likely to undergo nonsense-mediated decay. Segregation testing for the RP105 family was carried out in three affected and three phenotypically normal family members, and the mutation co-segregated with all patients (Figure 4C and Supplementary Figure S1).

The affected individual of family RP106 carried a known homozygous missense variant, i.e., c.547C>T; p.Leu183Phe in the *NMNAT1* gene (Figure 4D). This variant is likely to be pathogenic as (1) it is rare in population and is only observed once in the gnomAD database with an estimated frequency of 0.0004%; (2) the variant is conserved from chicken to human; (3) multiple in silico prediction programs suggested it is deleterious with a CADD score of 22. Consistently, segregation testing in four affected and five unaffected members of the family showed that this variant co-segregates with the disease phenotype (Figure 4D and Supplementary Figure S1).

A novel homozygous variant in exon 6 in the *EYS* gene, i.e., c.5571_5576delinsCTAGAT: p.Leu1858*, that leads to a premature stop codon was identified in RP107 (Figure 4E). This variant is likely to be pathogenic as the early termination removes approximately 40% of the protein, including all the laminin G-like domains. Alternatively, due to the premature stop codon, the mRNA may undergo nonsense-mediated decay. This variant is rare in population, and it has not been observed in the gnomAD database. Segregation of the mutation with the disease is observed for this family by genotyping four affected and three unaffected family members, further supporting the pathogenicity of this variant (Figure 4E, Supplementary Figure S3A).

One homozygous previously reported large deletion, i.e., c.9911_11550del, resulting in loss of four exons (exons 13–16) causing p.Asn3306Lys*7 was identified in the *ALMS1* gene in family RP109. This deletion was validated by performing PCR testing using a pair of primers adopted from Nikopoulos et al., 2015 (Supplementary Figure S2c) [26] and four primer pairs, one for each of exon 13–16 (Supplementary Table S2). These PCRs confirmed the exact break points of the deleted sequence (chr2: 73,772,326–73,813,432, Supplementary Figure S2c) as well as failure to obtain the band of the targeted region with patients’ DNA as a template confirmed segregation of mutation (Supplementary Figure S2b).This variant has not been observed in the gnomAD database and is pathogenic according to ACMG classification. The affected patient in RP110 carried a known heterozygous frameshift mutation, i.e., c.109del; p.Ala37Profs*17 in PAX6 gene (Figure 4F). This mutation is absent from the gnomAD database. Segregation testing for the family was carried out for two affected and two unaffected family members, confirming the segregation of the mutant allele with the disease phenotype (Figure 4F, Supplementary Figure S3B).

The same homozygous insertion that leads to a novel frameshift mutation, i.e., c.471dup causing p.Pro158Alafs*39 in exon 5 of the *SPATA7* gene, was found in two families, i.e., RP112 and RP113 (Figure 4G). This frameshift mutation has not been observed in the gnomAD database. A segregation test was carried out in families RP112 and RP113 for four affected and their parents. All affected members are homozygous for the mutation, while unaffected parents are carriers of the mutant allele (Figure 4G, Supplementary Figure S3C). The affected individuals of both families had severe phenotypes as all affected were suffering from LCA by birth. The age of affected individuals ranged from 5 to 50 years at the time of enrollment for this study. The youngest affected individual (IV.VIII) of RP113 was five years old at the time of enrollment for this study and had bilateral blindness with nystagmus.

## 4. Discussion

Pakistan has one of the highest rates of inherited genetic diseases in the world, consistent with the prominence of consanguinity within the society, harboring one of the highest rates of blindness and vision impairment as well. However, despite the high prevalence of IRDs in the population, limited studies on the genetic spectrum of IRDs have been undertaken in the region, likely because of underdevelopment, socio-economic limitations and lack of proper medical infrastructure or resources in the country. Furthermore, several studies in recent years indicate that a large portion of mutations were highly specific to families of Pakistani descent [33]. Therefore, the data represented in this study provides important insight into the genetic landscape of IRDs in Pakistan, providing valuable resources for both affected populations residing in the region and medical researchers from around the world. Results of this study will assist with future research of patients in this region, instigating the application of gene therapy techniques to assist families seeking treatment in this relatively underdeveloped country.

The data presented in this study identified the disease-causing genetic variants in a cohort of nine Pakistani families. A total of eight distinct pathogenic variants in seven genes were identified, including two missense, three nonsense, one frameshift indel and one deletion allele (Figure 4, Supplementary Figures S2 and S3). There is significant mutation heterogeneity within the population, considering that only the *SPATA7* and *USH2A* genes were repeated in more than one family. This high heterogeneity within the cohort urges extensive studies involving Pakistani families using comprehensive molecular diagnostic approaches to further explore genetic components to form a better understanding of disease mechanisms and identify founder mutations for the development of suitable treatment options [34].One interesting observation from this study was that two families carry the same novel SPATA7 pathogenic mutations, in contrast to the general reported rarity of mutations in *SPATA7* as a cause of retinal dystrophy [35]. These data suggest a unique mutation spectrum of IRDs in the study population. In family RP102, other phenotypic abnormalities such as mental retardation and acute leukemia were also observed, suggesting the involvement of some other genetic variant running in the family (Supplementary Figure S1b).

Among the eight pathogenic mutations detected in this study, six of them have been reported. A variant c.304C>A: p.Arg102Ser in *PDE6A* detected in RP101 is rare and was previously reported by Maria et al., 2015, Ullah et al., 2016 and Khan et al., 2021 [28,36] in families from Pakistan. This variant has been reported as a pathogenic mutation as it has been observed in multiple patients with autosomal recessive retinitis pigmentosa [27,28,36,37]. The stop-gain variant c.187C>T: p.Arg63* in *USH2A* is common and has been observed in individuals with Usher Syndrome [29,38,39]. Previously, this variant has been reported from different regions of the world, i.e., China [37], Spain [38], Denmark [39] and Italy [40]. Another cytosine to adenine substitution, i.e., c.1560C>A (p.Cys520*) in *USH2A* identified in family RP 105 in this study, was also reported previously in the Spanish population by González-del Pozo et al.in 2018 as a heterozygous allele in a non-syndromic retinitis pigmentosa patient along with another heterozygous variant c.2276G>T: p.Cys759Phe. Previous studies also indicate that mutations in *USH2A* can result in a broadly variable clinical outcomes between patients varying from non-syndromic IRD to Usher phenotype [41]. The same observation is reported for the genes implicated in Bardet–Biedl syndrome. Interestingly, a frame shift mutation in *USH2A* at the same position (p.Cys520Alafs*71) has also been reported to be pathogenic [29,42,43,44].

A large deletion encompassing a ~41.1Kb region of the *ALMS1* gene, i.e., c.9911_11550del identified in family RP109, has been previously reported by Nikopouloset al. in 2015 in two affected siblings of a consanguineous Pakistani family [26]. Interestingly, the family reported by Nikopouloset al. in 2015 and RP109 both belonged to the KPK province of Pakistan but are unrelated. Upon comparison of disease phenotype, it was found that affected members of RP109 also had acanthosis nigricans, obesity, vision and hearing loss since early childhood; however, we could not get them tested for renal function, cardiomyopathy and hepatomegaly as patients refused to cooperate. According to the Human Genome Mutation Database (http://www.hgmd.cf.ac.uk (accessed on 1 September, 2022)), there are 10 reported large deletions in the *ALMS1* gene, among which three include deletion of region comprising exons 13–16. Monzo et al. (2017) identified a 38Kb deletion comprising exon 13-16 of the *ALMS1* gene in a Pakistani female Alstrom-syndrome-affected case [45]. They used Sanger’s sequencing and the SNPs/CNVs microarray approach to define the exact location of deleted nucleotides, as well as showed that a long contiguous stretch (8.24 Mb) of homozygosity is centered in *ALMS1* sequence from the Pakistani population. According to Monzoet al.’s(2017) findings, the deletion of exons 13-16 is fixed in Pakistani recurrent haplotype, specifically in cases from northern Pakistani areas, i.e., KPK [45], and our findings support this notion.

The heterozygous variant, i.e., c.109del leading to p.Ala37Profs17 detected in exon 5 of *PAX6* gene in family RP110, is reported to cause nonsense-mediated decay [46]. This disease-causing variant has previously been reported as causative of aniridia [47], and the same phenotype was detected in cases of the RP110 family. In the *PAX6* gene, most of the detected variants are in heterozygous condition that partially disrupts the protein, suggesting haplo-insufficiency is enough for loss of PAX6 function [48].

As 25% of the pathogenic variants identified in this study are novel, however, future studies using a large number of familial cases are required to reveal population-specific disease-causing variants. Given that known alleles are notably easier to interpret than novel alleles due to limitations from lack of data for the latter, it is a requisite to construct a substantial database for Pakistani cohorts to improve the ability of interpretation for future studies. It is evident that additional sequencing of this population is essential for the future expansion of research and genetic counseling in Pakistan.

## Figures and Tables

**Table 1:** Demographic and clinical data of nine inherited retinal dystrophies affected families enrolled for this study.

Family ID	Proband ID	Age of Onset	Age at Enrollment	No of Affecteds	Consanguinity	Family History	Disease Progression	Symptoms
**RP 101**	III.IV	By birth	41 years	5	YES	Positive	Stationary	Night blind
**RP102**	IV.I	9 years	40 years	3	YES	Positive	Progressive	Night blind, Hearing loss
**RP105**	V.IV	14 years	26 Years	3	YES	Positive	Progressive	Night blind, Myopia, Hearing loss
**RP106**	V.II	By birth	7 Years	6	YES	Positive	Stationary	Night blind, Uncontrolled body movements
**RP107**	IV.XIII	12 years	30 years	9	YES	Positive	Progressive	Night blind, Epiphora, Myopia, Blurred vision
**RP109**	IV.I	2–6 years	15 years	3	YES	Positive	Progressive	Night blind, Nystagmus, Hearing problem, Poor day vision
**RP110**	V.IV	By birth	32 years	5	YES	Positive	Progressive	Night blind, Photosensitive, Nystagmus, Poor day vision
**RP112**	V.VIII	By birth	45 years	4	YES	Positive	Progressive	Night blind, Maculopathy
**RP113**	IV.III	By birth	22 years	5	YES	Positive	Progressive	Night blind, Maculopathy

**Table 2:** List of previously reported and novel pathogenic variants identified in nine unrelated inherited retinal dystrophies families segregating with disease phenotype in recessive form.

Family ID	Disease	NM_ID	cDNA Change	Protein Change	Gene	Genotype Status	Allele Type	Reference	ACMG Prediction
**RP 101**	RP	NM_000440	c.304C>A	p.Arg102Ser	*PDE6A*	Homozygous	Known missense	[27,28]	Likely Pathogenic
**RP102**	USH	NM_206933	c.187C>T	p.Arg63 *	*USH2A*	Homozygous	Known stop-gain	[29]	Pathogenic
**RP105**	USH	NM_206933	c.1560C>A	p.Cys520 *	*USH2A*	Homozygous	Known stop-gain	[30]	Pathogenic
**RP106**	LCA	NM_022787	c.547C>T	p.Leu183Phe	*NMNAT1*	Homozygous	Known missense	rs1337014971	Likely Pathogenic
**RP107**	RP	NM_001142800	c.5571_5576delinsCTAGAT	p.Leu1858 *	*EYS*	Homozygous	Novel stop-gain	N/A	Pathogenic
**RP109**	AS	NM_015120	c.9911_11550del	p.Asn3306Lysfs *7	*ALMS1*	Homozygous	Known deletion	[26]	Pathogenic
**RP110**	Aniridia	NM_001258465	c.109del	p.Ala37Profs *17	*PAX6*	Heterozygous	Known frameshift	[31]	Pathogenic
**RP112**	LCA	NM_001040428	c.471dup	p.Pro158Alafs *39	*SPATA7*	Homozygous	Novel frameshift	N/A	Pathogenic
**RP113**	LCA	NM_001040428	c.471dup	p.Pro158Alafs *39	*SPATA7*	Homozygous	Novel frameshift	N/A	Pathogenic

* RP: Retinitis Pigmentosa, USH: Usher Syndrome, LCA: Leber’s Congenital Amaurosis, AS: Alstrom Syndrome, N/A: Not Available.

## Data Availability

All data relevant to the study are included in the manuscript.

## Supplementary Materials

The following supporting information can be downloaded at: https://www.mdpi.com/article/10.3390/genes13091630/s1, Figure S1: Pedigree drawings of inherited retinal dystrophy families in which known mutations were detected, i.e., RP101 RP102, RP105, RP106 and RP110 showing autosomal recessive pattern of phenotype. Squares and circles denote males and females, respectively. Filled symbols show affected, while unfilled symbols show unaffected individuals. Double lines indicate consanguineous union. m1/m1, m2/m2, m3/m3, m4/m4 and m5/m5 refer to the homozygous disease-causing variants c.304C > A, c.187C>T, c.1560C>A, c.547C>T and c.109del, respectively, whereas m/+and +/+ refer to heterozygous carrier and homozygous normal status. Figure S2: (a) Pedigree drawing of inherited retinal dystrophy family RP109 showing autosomal recessive pattern of phenotype. Squares and circles denote males and females, respectively. Filled symbols show affected, while unfilled symbols show unaffected individuals. m1/m1 refers to the homozygous disease-causing variant, whereas m1/+ refers to heterozygous carrier status. (b) Validation of deletion, i.e., c.9911_11550del found in RP109 in the *ALMS1* gene by PCR testing. Four pairs of primers for exonic region 13–16 were used. Four primer pairs, i.e., Exon 13, Exon 15, Exon 16-1 and Exon 16-2, failed to amplify the region in patient DNA due to this deletion, however, in positive control, both the DNA of the patient and wild-type individual were amplified, as shown in the last wells of the gel photograph. (c) Sanger sequencing results confirming the exact breakpoints of gross deletion, i.e., c.9911_11550del found in RP109 in the *ALMS1* gene. Figure S3: (A) Segregation testing for novel homozygous deletion variant c.5571_5576delinsCTAGAT: p.Leu1858* found in RP107 in four affected (i–iv) and three unaffected (v–vii) family members. (B) Segregation testing for heterozygous variant c.109del: p.Ala37Profs*17 found in RP110 in two affected (i,ii) and two unaffected (iii,iv) family members. (C) Segregation testing results for homozygous duplication c.471dup: p.Pro158Alafs*39 checked in RP113 in four affected (i–iv) and two unaffected (v,vi) family members. Table S1: List of 344 genes screened through targeted exome sequencing in this study. Table S2: A list of primers used to amplify ALMS1 gene exonic region (exon 13–16) to confirm gross deletion, i.e., c.9911_11550del in RP109.
