# Supplementary material for: Exome Sequencing Identified Molecular Determinants of Retinal Dystrophies in Nine Consanguineous Pakistani Families
Source: Genes (Basel). 2022 Sep 10;13(9):1630. doi: 10.3390/genes13091630 (PMC9498396; doi:10.3390/genes13091630)
Supplement: Supplementary file 1 [file genes-13-01630-s001.zip › genes-1779512-supplementary.pdf]

Supplementary Data

Original article:

Title: Exome sequencing identified molecular determinants of retinal dystrophies in nine consanguineous Pakistani families

Supplementary figures:

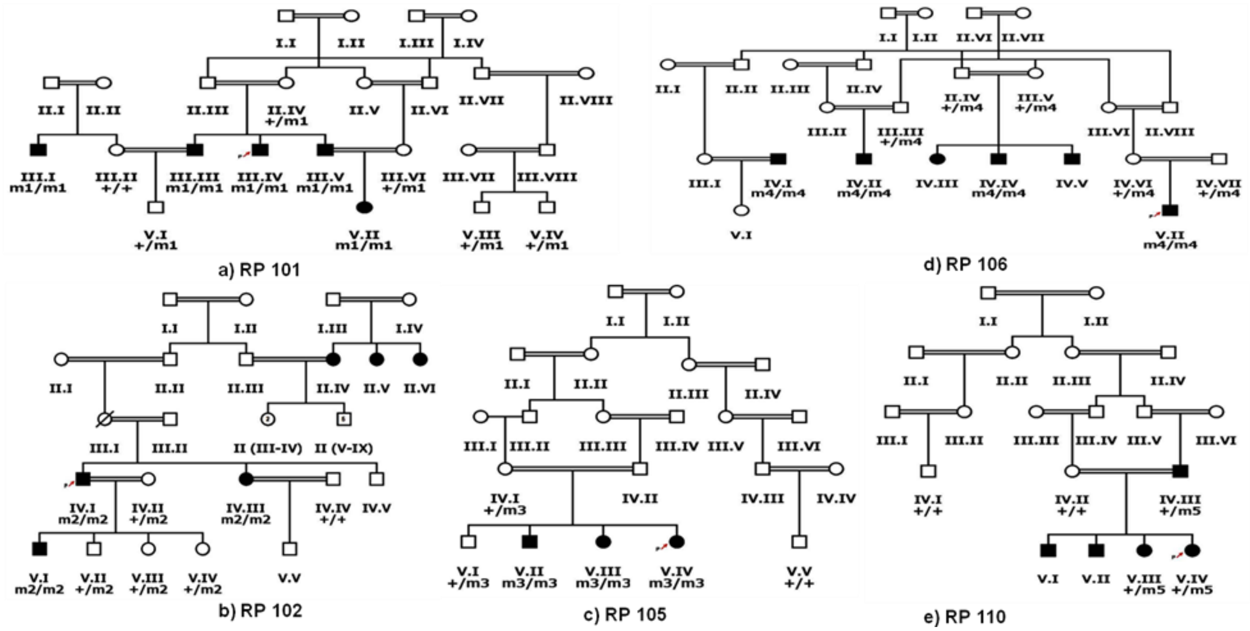

Supplementary figure S1: Pedigree drawings of inherited retinal dystrophy families in which known mutations were detected i.e., RP101 RP102, RP105, RP106 and RP110 showing autosomal recessive pattern of phenotype. Squares and circles denote males and females respectively. Filled symbols show affected individuals while unfilled symbols show unaffected individuals. Double lines indicate consanguineous union. m1/m1, m2/m2, m3/m3, m4/m4 and m5/m5 refer to the homozygous disease causing variants c.304C>A, c.187C>T, c.1560C>A, c.547C>T and c.109del respectively, whereas m/+and +/+ refer to heterozygous carrier and homozygous normal status.

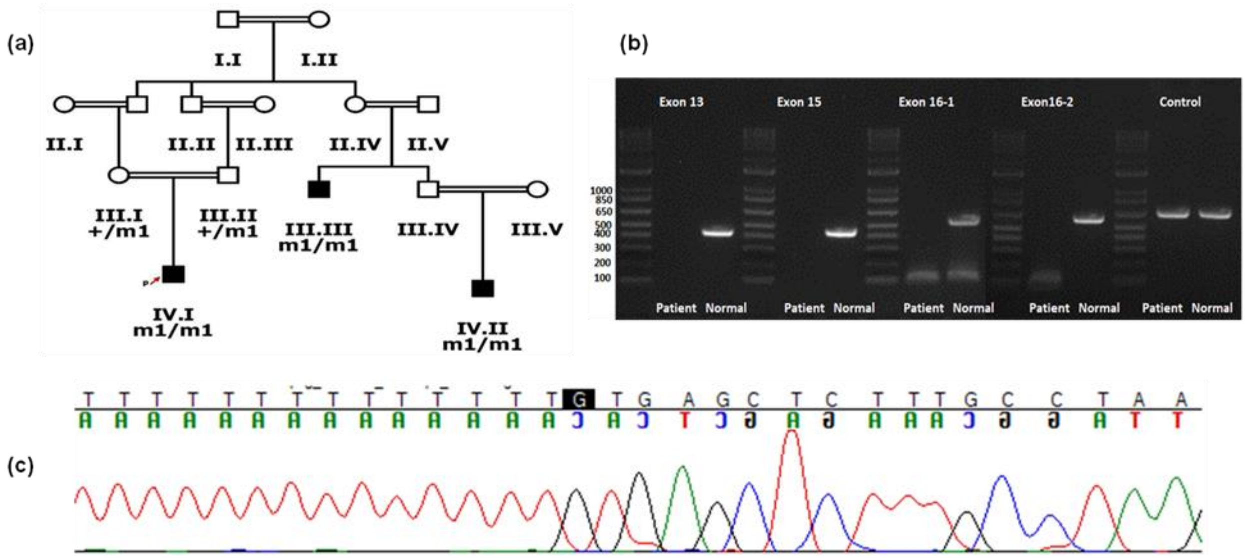

Supplementary figure S2: (a) Pedigree drawing of inherited retinal dystrophy family RP109 showing autosomal recessive pattern of phenotype. Squares and circles denote males and females respectively. Filled symbols show affected while unfilled symbols show unaffected individuals. m1/m1 refers to the homozygous disease causing variant whereas m1/+ refers to heterozygous carrier status. (b) Validation of deletion i.e., c.9911_11550del found in RP109 in *ALMS1* gene by PCR testing. Four pairs of primers for exonic region 13-16 were used. Four primer pairs i.e., Exon 13, Exon 15, Exon 16-1, and Exon 16-2 failed to amplify the region in patient DNA due to this deletion, however in positive control both the DNA of patient and wild type individual got amplified as shown in the last wells of gel photograph. (c) Sanger sequencing results confirming the exact breakpoints of gross deletion i.e., c.9911_11550del found in RP109 in *ALMS1* gene.

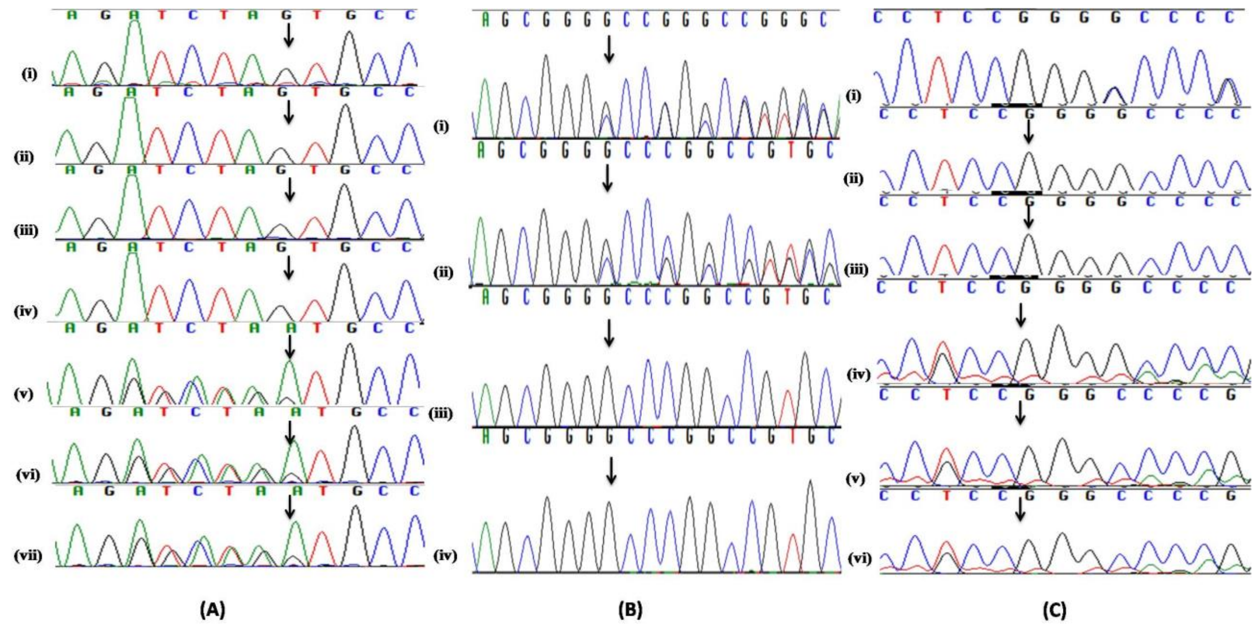

Supplementary figure S3: (A) Segregation testing for novel homozygous deletion variant c.5571_5576delinsCTAGAT: p.Leu1858* found in RP107 in four affected (i, ii, iii, iv) and three unaffected (v, vi, vii) family members. (**B)** Segregation testing for heterozygous variant c.109del: p.Ala37Profs*17 found in RP110 in two affected (i, ii) and two unaffected (iii, iv) family members. (**C)** Segregation testing results for homozygous duplication c.471dup: p.Pro158Alafs*39checked in RP113 in four affected (i, ii, iii, iv) and two unaffected (v, vi) family members.

Supplementary table S1: List of 344 genes screened through targeted exome sequencing in this study

ABCA4	*ARL6*	*BEST1*	*CDH23*	*CLN6*
ABCC6	*ASIC2*	*C12orf65*	*CDH3*	*CLN8*
ABHD12	*ASIC3*	*C1QTNF5*	*CDHR1*	*CLRN1*
ACBD5	*ATF6*	*C21orf2*	*CEP164*	*CLUAP1*
ADAM9	*ATOH7*	*C2orf71*	*CEP290*	*CNGA1*
ADAMTS18	*ATP1B2*	*C5orf42*	*CEP41*	*CNGA3*
ADGRA3	*ATXN7*	*C8orf37*	*CERKL*	*CNGB1*
ADGRV1	*BBIP1*	*CA4*	*CFH*	*CNGB3*
AGTPBP1	*BBS1*	*CABP4*	*CHM*	*CNNM4*
AHI1	*BBS10*	*CACNA1F*	*CIB2*	*COL11A1*
AIFM1	*BBS12*	*CACNA2D4*	*CISD2*	*COL2A1*
AIPL1	*BBS2*	*CAPN5*	*CLCN2*	*COL9A1*
ALMS1	*BBS4*	*CC2D2A*	*CLCN3*	*CRB1*
ARL13B	*BBS5*	*CCDC66*	*CLCN7*	*CRB2*
ARL2BP	*BBS7*	*CCL2*	*CLN3*	*CROCC*
ARL3	*BBS9*	*CCR2*	*CLN5*	*CRX*

CSPP1	*FAM161A*	*GRM6*	*INVS*	*LZTFL1*

CTSD	*FBLN5*	*GUCA1A*	*IQCB1*	*MAK*
CTSF	*FLVCR1*	*GUCA1B*	*ITM2B*	*MCOLN1*
CYP4V2	*FSCN2*	*GUCY2D*	*JAG1*	*MDM1*
DFNB31	*FZD4*	*GUCY2F*	*KCNJ13*	*MERTK*
DHDDS	*GBF1*	*HARS*	*KCNV2*	*MFN2*
DHX38	*GDF6*	*HK1*	*KIAA1549*	*MFRP*
DMD	*GJA10*	*HMCN1*	*KIF11*	*MFSD8*
DNAJC5	*GNAT1*	*IDH3B*	*KIF7*	*MITF*
DTHD1	*GNAT2*	*IFT140*	*KIZ*	*MKKS*
EFEMP1	*GNGT1*	*IFT172*	*KLHL7*	*MKS1*
ELOVL4	*GNPTAB*	*IFT27*	*LCA5*	*MPP5*
EMC1	*GNPTG*	*IMPDH1*	*LPCAT1*	*MTTP*
ERCC6	*GPR125*	*IMPG1*	*LRAT*	*MVK*
ERCC8	*GPR179*	*IMPG2*	*LRIT3*	*MYO7A*
EYS	*GRK1*	*INPP5E*	*LRP5*	*NDP*
NEK2	*NPHP1*	*NR2E1*	*NRL*	*NYX*
NEUROD1	*NPHP3*	*NR2E3*	*NXNL1*	*OAT*
NMNAT1	*NPHP4*	*NR2F1*	*NXNL2*	*OFD1*
OPA1	*PEX1*	*PITPNM3*	*RD3*	*RPGR*

OPA3	*PEX10*	*PLA2G5*	*RDH11*	*RPGRIP1*
OPN1LW	*PEX11B*	*POMGNT1*	*RDH12*	*RPGRIP1L*
OPN1MW	*PEX12*	*PPT1*	*RDH5*	*RRAS2*
OPN1SW	*PEX13*	*PRCD*	*RDH8*	*RS1*
OTX2	*PEX14*	*PRKCZ*	*REEP6*	*SAG*
PANK2	*PEX16*	*PROM1*	*RGR*	*SDCCAG8*
PAX2	*PEX19*	*PRPF3*	*RGS9*	*SEMA4A*
PAX6	*PEX2*	*PRPF31*	*RGS9BP*	*SLC24A1*
PCDH15	*PEX26*	*PRPF4*	*RHO*	*SLC38A8*
PCYT1A	*PEX3*	*PRPF6*	*RIMS1*	*SLC4A7*
PDCL	*PEX5*	*PRPF8*	*RLBP1*	*SLC6A6*
PDE6A	*PEX6*	*PRPH2*	*ROM1*	*SLC7A14*
PDE6B	*PEX7*	*RAB28*	*RP1*	*SNRNP200*
PDE6C	*PFDN5*	*RAX2*	*RP1L1*	*SPATA7*
PDE6G	*PGK1*	*RB1*	*RP2*	*SRD5A3*
PDE6H	*PHYH*	*RBP3*	*RP9*	*TCTN1*
PDZD7	*PIN1*	*RBP4*	*RPE65*	*TCTN3*
TEAD1	*TIMM8A*	*TIMP3*	*TMEM126A*	*TMEM138*
TMEM216	*UNC119*	*MIR204*	*NBAS*	*PROX1*

TMEM231	*USH1C*	*PLK4*	*PRDM13*	*BMP2*
TMEM237	*USH1G*	*PNPLA6*	*RTN4IP1*	
TMEM67	*USH2A*	*POC1B*	*SLC25A46*	
TOPORS	*VCAN*	*PRPS1*	*SPP2*	
TPP1	*VLDLR*	*TUBGCP4*	*TRNT1*	
TREX1	*VPS13B*	*GPR143*	*FBN1*	
TRIM32	*WDPCP*	*PLA2G6*	*CEP78*	
TRPM1	*WDR19*	*TRAF3IP1*	*CWC27*	
TSPAN12	*WFS1*	*C10orf11*	*ZGPAT*	
TTC21B	*ZNF408*	*TYR*	*IDH3A*	
TTC8	*ZNF423*	*APOB*	*IFT81*	
TTLL5	*ZNF513*	*AGBL5*	*FRMD7*	
TTPA	*CEP250*	*ADIPOR1*	*MC4R*	
TUB	*DRAM2*	*CTNNA1*	*MSX2*	
TUBGCP6	*HGSNAT*	*EXOSC2*	*BMP4*	
TULP1	*HMX1*	*GNB3*	*SF3B4*	
UCHL3	*LAMA1*	*MAPKAPK3*	*PITX1*	

Supplementary table S2: A list of primers used to amplify *ALMS1* gene exonic region (13-16) deletion of c.9911_11550del in RP109

**Exon No**	**Primer**	**Sequence**

**Exon 13**	Forward	CTCGTGTAAAACGACGGCCAGTCATAGAATTGGTCTAAGAGGCAAA
	Reverse	CTGCTCAGGAAACAGCTATGACATGCTCAATATAACAGCAAGGAGA
**Exon 15**	Forward	CTCGTGTAAAACGACGGCCAGTCCGCTACCTCTTTTTCTGACTG
	Reverse	CTGCTCAGGAAACAGCTATGACACCCAATCCCATTACCTTCAA
**Exon 16-1**	Forward	CTCGTGTAAAACGACGGCCAGTCTACCCGTTCTGTCTTCAGGTC
	Reverse	CTGCTCAGGAAACAGCTATGACTGAGACCTGGAGAGAATGTGTG
**Exon 16-2**	Forward	CTCGTGTAAAACGACGGCCAGTACAAAGGGATCAGAAGGTCACC
	Reverse	CTGCTCAGGAAACAGCTATGACATTCGACAGTAGAAGTGGTGCC
